# BMSCs promote the differentiation of NSCs into oligodendrocytes via mediating Id2 and Olig expression through BMP/Smad signaling pathway

**DOI:** 10.1042/BSR20180303

**Published:** 2018-09-21

**Authors:** Peiwen Song, Xiang Xia, Tianyu Han, Huang Fang, Ying Wang, Fulong Dong, Renjie Zhang, Peng Ge, Cailiang Shen

**Affiliations:** 1 Department of Orthopedics (Spinal Surgery), The First Affiliated Hospital of Anhui Medical University, No.218 Jixi Road, Shushan District, Hefei City, Anhui Province, China; 2 Department of Medical Imaging, The First Affiliated Hospital of Anhui Medical University, No.218 Jixi Road, Shushan District, Hefei City, Anhui Province, China

**Keywords:** bone morphogenetic protein, mesenchymal stem cell, neural stem cells, spinal cord injuries

## Abstract

Neural stem cells (NSCs) have emerged as a promising treatment for spinal cord injuries. However, the increasing expression of bone morphogenetic proteins (BMPs) in spinal cord injury lesion sites seems to have contributed to the limited oligodendroglial differentiation and the majority of the astroglial differentiation of NSCs. In the present study, we demonstrate that BMPs promote NSCs differentiation toward astrocytes and prevent them from differentiating into oligodendrocytes. This effect is accompanied by the increasing expression of Id2 and the reduction in Oilg1/2 expression. Treatment with bone marrow stromal cells (BMSCs) can enhance the development of oligodendrocytes in the presence of BMPs. The analysis of Id2, as well as Olig1 and Olig2 gene expression, reveals that the effect of BMPs on these gene expressions is reversed with the addition of BMSCs. In sum, these data strongly suggest that BMSCs can promote the differentiation of NSCs into oligodendrocytes through mediating Id2 and Olig1/2 expression by blocking the BMP/Smad signaling pathway.

## Introduction

Spinal cord injury (SCI) is caused by direct mechanical damage to the spinal cord that induces neural cells apoptosis and demyelination of axons resulting in severe neurological functional disability that affects sensory function and mobility [1]. Therefore, most studies have focused on treatments to promote neural regeneration or to replace lost neurons and neural cells [2,3].

Neural stem cells (NSCs) are capable of generating all three major central nervous system lineages (neurons, oligodendrocytes, and astrocytes) and seem to contribute to their self-repair after a SCI by replacing lost neural cells and trophic support [4,5]. Moreover, a spinal cord injury induces the activation of the endogenous NSCs and leads to the robust expansion of these cells [6,7]. However, the effect of this spontaneous repair is inadequate because of limited oligodendroglial differentiation and the fact that a majority of the cells differentiate into astrocytes, which contributes to glial scar formation [8]. Thus, more researchers have realized that some extrinsic factors that increase or produce in a spinal cord lesion site, might be key to determining the fate of endogenous NSCs.

One particularly interesting factor is bone morphogenetic proteins (BMPs). BMPs are secreted factors, and the expression of BMPs is significantly increased in the lesion site. *In vitro* and *in vivo* studies have demonstrated that BMPs could promote the differentiation of astrocytes and inhibit oligodendroglial lineage specification in NSCs [9–12]. Therefore, further studies are starting to focus on the suppression of the expression of BMPs after SCI [13,14]. One of the most interesting studies has focused on mesenchymal stem cells (MSCs) [15].

MSCs are a type of self-renewing and multipotent stem cell that was first found in bone marrow [16,17]. Because of the ability of MSCs to be differentiated into three major neural cells, initial research has focused on transplanting MSCs in SCI animal models to replace the lost neural cells in the lesion site [18–21]. Several studies have reported improvements in neurological function [22–24]. After transplanting MSCs into an injured spinal cord, some researchers have documented inefficiency in the neuronal differentiation of MSCs and a lack of expression of neuronal markers in the lesion site [25,26]. Moreover, Gu et al. [27] reported that despite a lack of expression of oligodendrocyte cell markers in an injured spinal cord lesion site, the increasing number of axons was confirmed by a transmission electron microscopic examination. These results suggest that the therapeutic effects of MSC transplantation on neurological improvement might not be achieved by replacing the lost neural cells but by providing a favorable environment for endogenous NSC regeneration and the neural differentiation of NSCs. This includes the suppression of BMP expression at the lesion site. *In vitro* and *in vivo* treatment of bone marrow stromal cells (BMSCs) could markedly reverse the effect of BMP on the differentiation of NSCs and accelerate remyelination in an SCI lesion. Although the benefit of BMSCs on NSCs has been shown in studies, the molecular mechanisms responsible for this effect on an SCI have not been fully understood.

In the present study, we reveal that a BMP signaling pathway was able to promote astroglial differentiation of NSCs by mediating the expression of Id2 and Olig1/2. The effects of BMP4 were blocked by the treatment of BMSC-conditioned media (BMSC-CM). These data support the notion that a central role of MSCs is to provide a favorable environment by reversing the effect of BMPs on the expression of Id2 and Olig1/2 genes to stimulate the oligodendroglial differentiation of NSCs.

## Methods

### BMSC culture and CM preparation

BMSCs were harvested from the bone marrow of femurs and tibias of 3- to 4-month-old female Fisher 344 rats. Cells were seeded at 1 × 10^6^ cells/cm^2^ and expanded in α-minimum essential medium (α-MEM, Gibco Invitrogen) supplemented with 10% fetal bovine serum (FBS). After 3 days, the medium was changed, and the non-adherent cells were removed. Adherent cells were cultured with α- MEM-10% FBS until the cells were confluent. The cells were trypsinized by using 0.25% Trypsin (Gibco Invitrogen) and seeded in α-MEM-10% FBS at 8000 cells/cm^2^. The medium was changed every 3 days, and the cells were passaged when 90% of confluence was reached [28].

BMSC-CM was collected as follows: passage 3 BMSCs were seeded at 12,000 cells/cm^2^ with α-MEM-10% FBS until the cells were 90% confluent. The cells were washed three times with phosphate-buffered saline (PBS), and the medium was changed to a serum-free DMEM culture medium for 48 h. CM from different flasks were collected and pooled; then the CM were concentrated by centrifugation at 4000 ***g*** for 15 min at 13°C, using 10 k-Da MW filter units (Millipore). Next, we filtered the CM using 0.22 mm filters (Millipore) and stored the CM at −80°C [29].

### Isolation and culture of NSCs

The cultures and isolation of NSCs were mainly according to the previous studies [30,31]. NSCs were isolated from the cerebral cortex of 1- to 2-day-old newborn SD rats. The meninges were carefully removed. The brains were chopped into pieces and placed in Petri dish with ice-cold Hank’s balanced salt solution (Gibco, Invitrogen). The cells were expanded as neurospheres in a proliferation medium containing DMEM/F12, with 2% B27 supplement, epidermal growth factor (20 ng/ml), and basic fibroblast growth factor (10 ng/ml) for 7 days. The cells were passaged weekly. Neurosphere cultures from 2 to 3 were used throughout the present study.

### Exposure of NSCs to BMP4 or Noggin or BMSC-CM or both BMP4 and BMSC-CM

NSCs were plated on polyornithine and laminin-coated glass coverslips at a density of 1000 cells per cm^2^ in α-MEM-5% FBS for 24 h. After 30 min, 20 ng/ml BMP4, 1 ml of BMSC-CM with 5% FBS or 20 ng/ml BMP4 with 200 ng/ml Noggin, or 1 ml of BMSC-CM with 20 ng/ml BMP4 and 5% FBS were added to the medium of the wells. The cells were co-cultured for 7 days, and the medium was replaced on the third day. The cells were fixed for 30 min with phosphate-buffered 4% paraformaldehyde (37°C, pH 7.4) and processed for immunofluorescent labeling.

### Immunocytochemistry

The cells were fixed in 4% paraformaldehyde (Sigma) in 1× PBS for 20 min, washed three times in PBS, and then blocked with 1× PBS containing 1% bovine serum albumin and 0.25% Triton X-100. The same solution was used during the incubations with the primary antibodies. The cells were incubated with antibodies overnight at 4°C. Fluorochrome-conjugated secondary antibodies were used for immunodetection. The primary antibodies were mouse antiglial fibrillary acidic protein (anti-GFAP) 1:1000 (Abcam) and rabbit anti-Galactocerebroside (anti-GalC) 1:500 (Abcam). The secondary antibodies were donkey anti-mouse conjugated with Alexa Fluor 568 1:1000 (Invitrogen); donkey anti-rabbit conjugated with Alexa Fluor 488 1:1000 (Dianova). Nuclear counterstaining was performed with 4′,6′-diamidino-2-phenylindole dihydrochloride hydrate at 0.25 μg/ml (Sigma). Coverslips were mounted onto glass slides using a Prolong Antifade kit (Invitrogen). The cells were quantified and photographed using an Olympus IX81 fluorescent microscope equipped with a Hamamatsu digital camera. For each culture condition, ten randomly selected fields were photographed, and the frequency of selected cellular markers was determined for every condition in three independent experiments.

### RNA extraction and quantitative PCR

The total RNA from the cultured cells was extracted with Trizol reagent (Gibco, Life Technologies, Grand Island, NY, U.S.A.), and the total RNA was prepared according to the manufacturer’s instructions. The cDNA was synthesized by using Superscript III RT Reaction Mix (Invitrogen). Quantitative PCR (qPCR) was performed in a Realplex2 Mastercycler (Eppendorf). The Gene-specific primers are listed in Table 1. All reactions were performed with Sybr-Green master mix (Applied Biosystems) using the following cycling parameters: 95°C, 15 s and 60°C, 60 s for 40 cycles.

**Table 1:** Primer sequences

Gene names	Forward primer	Reverse primer
Id-2	TTTCCTCCTACGAGCAGCAT	CCAGTTCCTTGAGCTTGG AG
Olig-1	GCCCCACCAAGTACCTGTCTC	GGGACCAGATGCGGGAAC
Olig-2	CACAGGAGGGACTG TGTCCT	GGTGCTGGAGGAAGATGACT
GAPDH	ACCACAGTCCATGCCATCAC	TCCACCACCCTGTTGCTGTA

### Western blot assay

After a PBS wash, the cells were extracted in cold lysis buffer (20 mM Tris HCl, 150 mM NaCl, 1% Triton X-100, 0.5% sodium deoxycholate, 0.1% SDS, 2 mM EDTA, 1.0 mM sodium orthovanadate, 50 mM sodium fluoride, 200 mM phenylmethanesulfonyl fluoride, and 40 ml/ml of protease inhibitor cocktail). The proteins were separated by 12% acrylamide gel electrophoresis and transferred to a PVDF membrane. The member was blocked for 1 h in 10% skim milk at room temperature. The membranes were incubated overnight at 4°C with antibodies to Map-2 (1:2000, Abcam), GalC (1:2000, Abcam), Samd1 (1:1000, Signaling Technology, Danvers, MA, U.S.A.), and Smad5 (1:1000, Cell Signaling Technology, Danvers, MA, U.S.A.). Next, the blots were washed and incubated with a secondary antibody (Santa Cruz Biotechnology; 1:2000 in blocking solution) for 1 h at room temperature. After being washed three more times with TBS-T, the blots were developed using SuperSignal West Pico enhanced chemiluminescence reagent (Thermo Scientific).

### Statistical analysis

All the results were presented as mean ± standard error of the mean. Statistical analysis was performed using one-way analysis of variance (ANOVA), and the least significant difference method (LSD) was used for comparisons among multiple groups. Statistical analysis was performed using SPSS 16.0 software (Chicago, IL, U.S.A.). *P*<0.05 was considered significant.

## Results

### BMP4 promotes astroglial differentiation of NSCs

To determine the effect of BMP4 on the differentiation of NSCs, we incubated NSCs for 7 days in a control medium (α-MEM-5% FBS), in 20 ng/ml BMP4, or in 20 ng/ml BMP4 with Noggin (the BMP antagonist).

**Figure 1:**
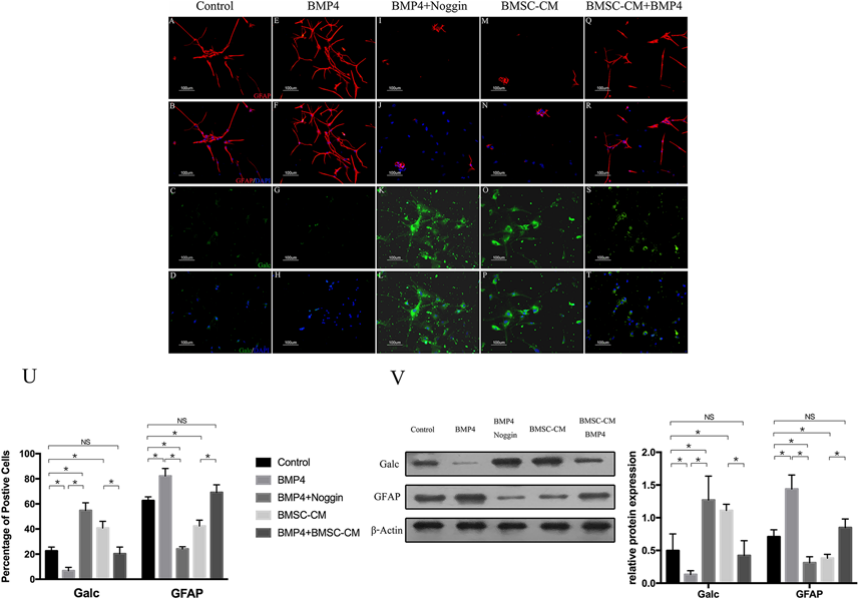
The proportions of GFAP^+^ and GalC^+^ cells were altered in the presence of BMP4 and reversed by Noggin or BMSC-CM (**A–D**) The proportions of GFAP^+^ and Galc^+^ cells in control groups (NSCs+5% FBS-α-MEM). (**E–H**) BMP4 promoted astroglial differentiation and inhibited oligodendroglial differentiation of NSCs. (**I–L**) The effect of BMP4 on the differentiation of NSCs was blocked by Noggin. (**M–P**) The GalC-expressing cells were increased, and the GFAP-expressing cells were decreased in the presence of BMSC-CM. (**Q–T**) The astroglial effect of BMP4 on NSCS was partly reversed by BMSC-CM. (**U**) Quantification of cell types in these five groups. The percentage of cells expressing GFAP. GalC was determined from 500 to 1000 cells in randomly chosen fields (one-way ANOVA for Galc expression: *F* = 79.291, *P*<0.001, df1 (between Groups)/df2 (within groups) = 4/20, *n*=5; one-way ANOVA for GFAP expression: *F* = 121.88, *P*<0.001, df1/df2 = 4/20, *n*=5; *P<0.05; NS = *P*>0.05). (**V**) Western blot showed the expression of GFAP and GalC in these five groups. The Western blot results were consistent with those of immunocytochemistry. The BMP4 groups had the highest expression of GFAP and the lowest expression of GalC, whereas the groups with Noggin had the lowest expression of GFAP and the highest expression of GalC (one-way ANOVA for Galc expression: *F* = 13.30, *P*=0.01, df1/df2 = 4/10, *n*=3; one-way ANOVA for GFAP expression: *F* = 14.18, *P*<0.001, df1/df2 = 4/10, *n*=3; *P<0.05; NS = *P*>0.05).

Consistent with the findings of previous studies, the percentage of GalC-expressing cells decreased significantly—from 23 ± 3% to 8 ± 3%—compared with that of the control group after incubation with NSCs (Figure 1A,B,E,F,U). In contrast, the percentage of GFAP-expressing cells increased from 63 ± 3% to 82 ± 6% (Figure 1C,D,G,H,U).

Next, we examined whether blocking the BMP4 with BMP-antagonist Noggin would affect the differentiation of NSCs. As expected, after the addition of Noggin to the BMP4 groups, the number of GalC-expressing cells increased (55 ± 6%) and the expression of GFAP decreased (24 ± 2%) (Figure 1I–L and U).

### BMP4 regulation of ID/Olig was Smad mediated

**Figure 2:**
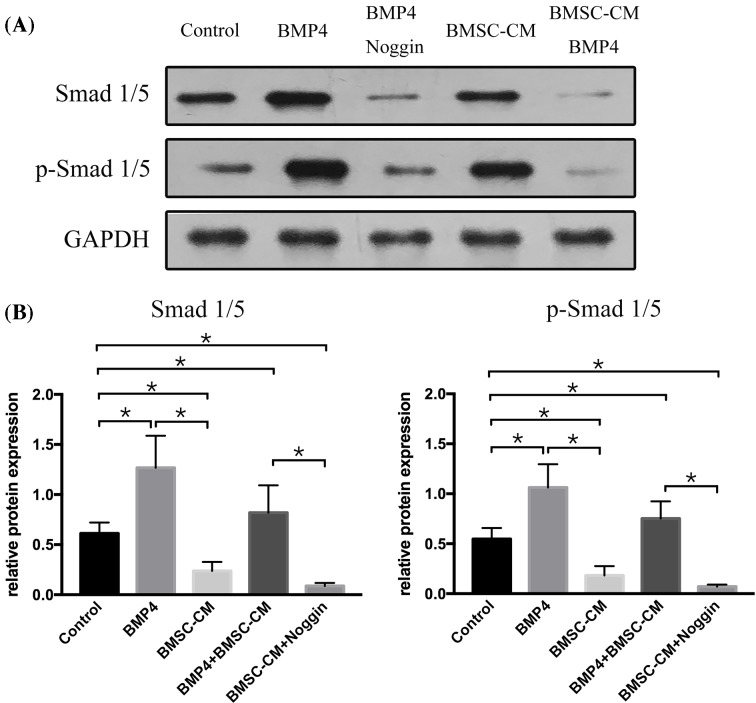
Smad1/5 is altered by BMP4 or BMSC-CM in NSCs The representative Western blot showed that the expression of Smad1/5 was up-regulated by BMP4 and blocked with the treatment of Noggin. In contrast, BMSC-CM alone could down-regulate the expression of Smad1/5, and it partly decreased the expression of Smad1/5 in the presence of BMP4. The data were expressed as mean ± SD (one-way ANOVA for Smad1 expression: *F* = 61.356, *P*<0.001, df1/df2 = 4/10, *n*=3; one-way ANOVA for Smad5 expression: *F* = 121.289, *P*<0.001, df1/df2 = 4/10, *n*=3; one-way ANOVA: *P<0.05; NS = *P*>0.05).

Consistent with the findings of previous studies, BMP4 promoted astroglial differentiation by activating the Smad1/5 signaling in NSCs. To identify the molecular mechanisms of BMP on NSCs, NSCs were cultured 7 days with BMP4 alone or in the presence of Noggin, with α-MEM-5% FBS serving as a control medium. Next, we evaluated the expression of Smad1/5 and p-Smad1/5 using a Western blot. The results showed that the expression of both Smad1/5 and p-Smad1/5 was significantly increased by the treatment of BMP4 on the cultured NSCs in comparison with the results obtained using the control medium. Moreover, this BMP4 up-regulation of Smad1/5 and p-Smad1/5 was completely prevented by the addition of Noggin (Figure 2).

**Figure 3:**
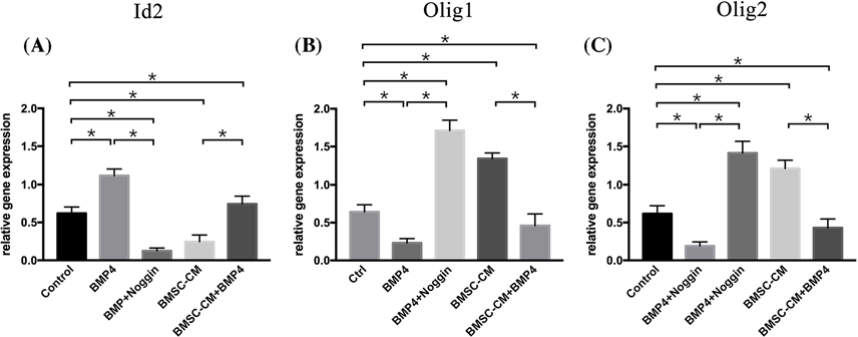
The effects of BMP4 or BMSC-CM were associated with changes in Id2, Olig1, and Olig2 expression Quantitative RT-PCR analysis of Id2, Olig1, and Olig2 expression revealed that cells treated with BMP4 showed the highest Id2 gene expression with the lowest Olig1 and Olig2 gene expression, whereas Noggin could block the effect of BMP4. It showed the lowest Id2 gene expression with the highest Olig1 and Olig2 gene expression. The cells incubated with BMSC-CM revealed a lower Id2 gene expression and a higher Olig1 and Olig2 gene expression compared with those of the control groups. BMSC-CM could partly counter the effect of BMP4 on the expression of Id2, Olig1, and Olig2 gene expression in NSCs. The relative expression of Id2, Olig1, and Olig2 was normalized to the housekeeping gene Glucose-6-phosphate dehydrogenase (G6PDH), and the data were expressed as mean ± SD (one-way ANOVA for Id2 expression: *F* = 49.562, *P*<0.001, df1/df2 = 4/10, *n* = 3; one-way ANOVA for Olig1 expression: *F* = 96.795, *P*<0.001, df1/df2 = 4/10, *n*=3; one-way ANOVA for Olig2 expression: *F* = 40.257, *P*<0.001, df1/df2 = 4/10, *n*=3; one-way ANOVA: *P<0.05; NS = *P*>0.05).

In the next set of experiments, we assessed the expression of oligodendrogenic transcription factors Olig1 and Olig2 and the inhibitor of differentiation (Id2). These factors were thought to be important for directing the oligodendrocyte/astrocyte fate determination of NSCs. Thus, NSCs were cultured for 7 days with BMP4 in the presence or absence of Noggin, and NSCs incubated with 5% FBS-α-MEM served as a control. The expression of Olig1 and Olig2 and Id2 were evaluated by quantitative RT-PCR. NSCs incubated in BMP4 showed the highest expression of Id2. In contrast, the expression level of Olig1 and Olig2 were lowest in this group. Moreover, this effect was completely reversed by the addition of Noggin to the BMP4 group. Id2 expression declined, whereas Olig1 and Olig2 expression increased (Figure 3). In sum, this suggests that the BMP4/Samd signaling pathway could direct the oligodendrocyte/astrocyte fate decision of NSCs by mediating Id/Olig gene expression.

### BMSC-CM could reverse the BMP-mediated effect in NSC differentiation from an astroglial to an oligodendroglial phenotype

To examine whether BMSC-CM could reverse the astrogenic effect of BMP4, NSCs were incubated for 7 days with BMSC-CM with or without BMP4. NSCs incubated in BMSC-CM showed a higher expression of GalC and a lower expression of GFAP compared with the control groups (Figure 1M–P and U). This oligodendroglial effect of BMSC-CM on NSCs was consisted with the findings of previous studies. By contrast, after exposure to BMSC-CM to NSCs in the presence of BMP4, the proportion of oligodendrocytes increased to 20 ± 5% and the percentage of astrocytes decreased to 69 ± 6% (Figure 1Q–T and U).

### BMSC-CM regulated Id/Olig via counter BMP/Smad signaling pathway

To investigate the molecular mechanism, we added BMSC-CM to NSCs in the presence of BMP4. Then, we first analyzed the expression of Smad1/5 and p-Smad1/5, the result showed that after the addition of BMSC-CM to BMP4 groups, the expression of Smad1/5 and p-Smad1/5 was decreased, as compared with that in BMP4 groups (Figure 2). Next, we examined the expression of Id2, as well as Olig1 and Olig2 in these groups (Figure 3). NSCs incubated with both BMSC-CM and BMP4 showed a lower Id2 expression and higher Olig1 and Olig2 expression compared with cells incubated with BMP4 alone.

**Figure 4:**
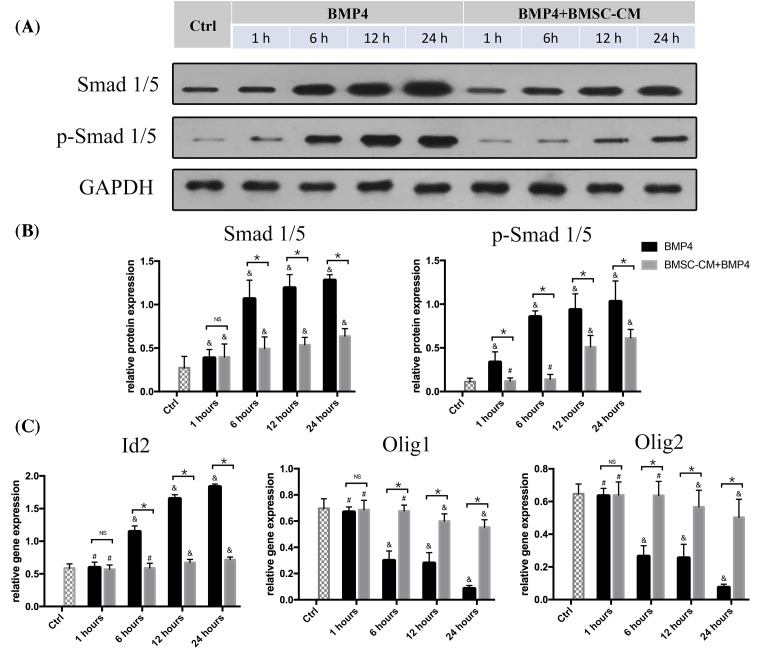
BMSC-CM reversed the effect of BMP4 on the mediation of the expression of Smad1/5, p-Smad1/5,Id2, Olig1, and Olig2 gene in NSCs (**A** and **B**) Western blot analysis showed that the expression of Smad1/5 and p-Smad1/5 was significantly increased after 6 h treatment with BMP4. The BMSC-CM treatment reduced levels of Smad1/5 and p-Smad1/5 expression in the presence of BMP4. (**C**) Expression of mRNAs for Id2, Olig1, and Olig2 was analyzed by quantitative RT-PCR with or without BMSC-CM in the presence of BMP4 for 1, 6, 12, and 24 h. The Id2 gene expression was increased and Olig1/2 was reduced by BMP4 at 6 h and subsequent time points. These changes from BMP4 treatment on NSCs were reversed by BMSC-CM (*n*=5, NS = *P*>0.05, *P<0.05, ^&^*P*<0.05 compared with control groups, ^#^*P*>0.05 compared with control groups).

To help define mechanisms underlying the effects of BMSC-CM on NSCs, we also examine the expression of Smad1/5, p-Smad1/5, Id2, Olig1, and Olig2 gene in the early time points (1, 6, 12, and 24 h) after exposure to BMP4 or both BMP4 and BMSC-CM. The results showed that Smad1/5 and p-Smad1/5 expression significantly increased with BMP4 treatment as early as 6 h. However, after the addition of BMSC-CM to NSCs in the presence of BMP4, only mild change in Smad1/5 expression was found, and the expression of p-Smad1/5 started to increase till 12 h after the exposure to both BMP4 and BMSC-CM. Moreover, the addition of BMSC-CM to BMP4 groups significantly reduced the expression of these proteins at 6 h and subsequent time points (Figure 4A,B). The quantitative RT-PCR analysis revealed that Id2 gene expression was significantly increased with the reduction in Olig1/2 gene expression at 6 h with the treatment of BMP4. In the co-culture groups, no significant increase in Id2 expression or reduction in Olig1 and Olig2 expression was observed after 6 h of BMP4+BMSC-CM treatment (*P*>0.05 compared with control groups). Only modest changes of these gene expressions were found after 12 h of co-culture compared with control groups (*P*<0.05). Similarly, the effect of BMP4 on the changes of Id2, Olig1, and Olig2 gene expression was reversed by the addition of BMSC-CM at 6 h and subsequent time points.

### The effect of BMSC-CM might not depend entirely on blocking the BMP signaling pathway

**Figure 5:**
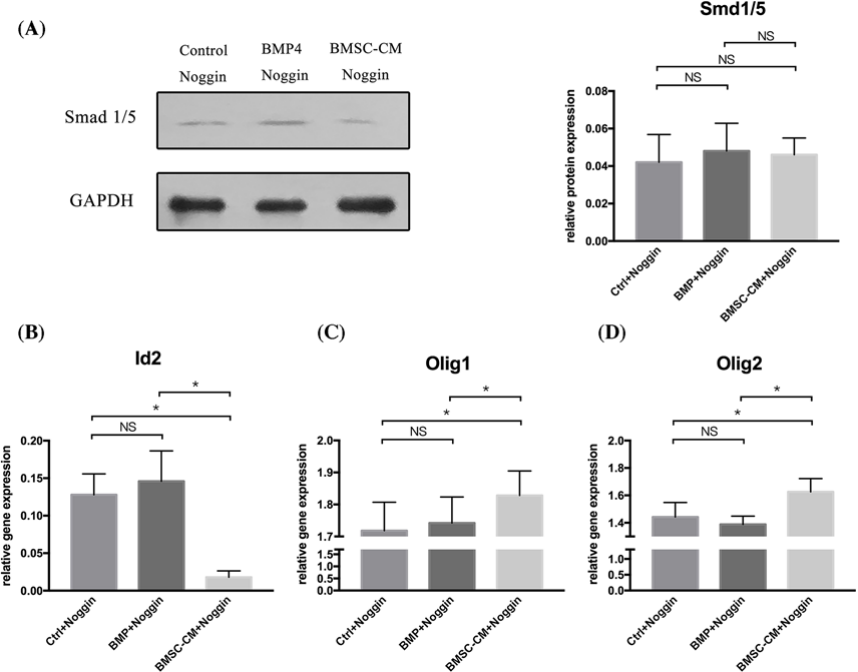
NSCs were incubated in 5% FBS-α-MEM, BMP4 or BMSC-CM–all in the presence of Noggin (**A**) Western blot analysis showed that the expression of Smad1/5 was significantly reduced by the addition of Noggin in these three groups. No statistical difference was found among these three groups (one-way ANOVA for Smad1/5 expression: *F* = 1.143, *P*=0.351, df1/df2 = 2/12, *n*=5. Control + Noggin vs BMSC-CM + Noggin: *P*=0.630; Control + Noggin vs BMP + Noggin: *P*=0.342; BMSC-CM + Noggin vs BMP + Noggin: *P*=0.163). (**B**) Quantitative RT-PCR analysis of Id2, Olig1, and Olig2 expression revealed that although the BMP/Smad pathway was completely blocked by Noggin, the expression of Id2, Olig1, and Olgi2 gene expression was still changed by BMSC-CM. No statistical difference was found between the Control and the BMP4 groups (one-way ANOVA for Id2 expression: *F* = 29.158, *P*<0.001, df1/df2 = 2/12, *n*=5; one-way ANOVA for Olig1 expression: *F*=8.972, *P*=0.04, df1/df2 = 2/12, *n*=5; one-way ANOVA for Olig2 expression: *F* = 9.623, *P*=0.03, df1/df2 = 2/12, *n*=5; one-way ANOVA: *P<0.05; NS = *P*>0.05).

During the present study, we also had an interesting finding. As the BMP/Smad signaling pathway played a key role in the regulation of the balance of Id2 and Olig1/2, BMSC-CM could regulate the balance through the BMP/Smad pathway. Theoretically, BMSC-CM might not exert its effect on NSCs if we completely blocked the BMP pathway. So, we used Noggin to block the BMP signaling pathway completely in control, BMP4, and BMSC-CM groups. Then, we investigated the expression of Id2 and Olig1/2 in these groups. The effectiveness of Noggin in blocking the BMP pathway was confirmed by examining the expression of Smad1 and Smad5 by a Western blot. This showed, for the most part, a reduction in the expression level of Smad1 and Smad5 in these three groups (Figure 5A). The analysis showed that the expression of Id2 and Olg1/2 was no different in the control and BMP4 groups. However, the BMSC-CM groups showed a lower expression of Id2 but a higher Olig1 and Olig2 expression compared with that of control and BMP4 groups (Figure 5B–D). This result revealed that although the BMP/Smad was completely blocked, the BMSC-CM could still have an effect on NSCs via other pathway. This indicates that the BMP/Smad pathway might not be the only pathway through which BMSC-CM mediate the Id and Olig balance of NSCs.

## Discussion

The present study demonstrates that BMPs enhance astroglial differentiation of NSCs by up-regulating Id2 expression and down-regulating Olig1/2 expression. BMSCs can reverse this effect of BMPs on NSCs and promote differentiation from an astroglial to an oligodendroglial phenotype. BMSCs exert this effect on NSCs by mediation of Id2, Olig1/2 gene expression via partly blocking the BMP/Smad signaling pathway.

The differentiating fate of NSCs plays a key role in neurological improvement after a spinal cord injury. Oligodendrocytes are essential for remyelination and axonal regeneration, which are important for the restoration of spinal cord circuitry. In contrast, the increase in astrocytes in the spinal cord lesion site was closely associated with the formation of a scar, which is considered detrimental to the recovery of axon and neurological improvement. Thus, the mediation of the glial balance of NSCs is very important for the treatment of a spinal cord injury.

Olig1 and Olig2 are Class B basic-Helix–Loop–Helix (bHLH) transcription factors that are critical for oligodendrogenesis [32,33]. In Olig1/Olig2 double-knockdown mice, a complete failure of oligodendrocyte development was shown in all areas of the brain, while a significant increase in astrocytes was found in the spinal cord [32]. Hwang et al. transplanted Olig2-NSCs into a contused spinal cord lesion site, compared with the group that received only NSC transplantation, this group achieved a better result with regard to locomotor recovery. More important, myelin sheath was observed. This was probably induced by the differentiation of NSCs into the oligodendrocytes [34]. Id2, which also belongs to the bHLH family of transcriptional factors, was involved in the oligodendrocyte/astrocyte fate decision of the NSCs as well [35,36]. Previous studies have shown that the up-regulation of Id2 could promote NSC differentiation from an astroglial to an oligodendroglial phenotype. Moreover, Id2 could directly sequester oligodendrogenic transcription factors Olig1 and Olig2, resulting in an increase in astrocytes and a reduction in oligodendrocytes [37]. Thus, the balance between the Olig1/2 and Id2 expression plays a crucial role in the oligodendrocyte/astrocyte fate decision of NSCs.

Our data were consistent with these studies [38–41]. The increase in the expression of Id2 could reduce the expression of Olig1/2 and promote NSC differentiation in astrocytes. In contrast, the reduction in Id2 expression induced the increase in Olig1 and Olig2, with the result that the differentiation of NSCs changed from an astroglial to an oligodendroglial phenotype.

After a spinal cord injury, BMPs are members of the transforming growth factor-b superfamily of pleiotropic growth factors and are up-regulated in the lesion site, inducing a massive endogenous NSCs differentiation into astrocytes. This, in turn, results in the formation of a glial scar and the failure of remyelination [42,43]. BMPs acquire their activity by binding to Type I and II BMP receptors to form a heterotetrameric complex. The signaling then phosphorylates the Smad proteins and forms complexes with the common mediator Smads, and this directly or indirectly regulates gene transcription, including that of Id genes [44,45]. Human renal proximal tubule epithelial cells use siRNAs to silence Smad expression. The results showed that BMP up-regulation of Id2 was significantly abolished by the Smad protein knockdown [46]. Thus, BMPs have been found to mediate the expression of Id genes in several cell types [47–49].

In sum, BMPs could regulate Id2 genes through Smad proteins. In our study, we found that both the Id2 level and the Smad1/5, p-Smad1/5 expression were up-regulated by BMP4 in NSCs. Moreover, this BMP4 induction of Id2 and Smad1/5, p-Smad1/5 was completely inhibited by the treatment of Noggin. These data were consistent with those of previous studies. Thus, BMP4 mediated the expression of Id2, Olig1, and Olig2 through the up-regulation of Smad proteins

BMSCs, which are a kind of self-renewing and multipoint stem cell, are favored in many studies on spinal cord injuries because they can be rapidly expanded and easily isolated. In addition, negative reactions to autologous or allogeneic transplantation have not been reported [50–52]. Although BMSCs are capable of generating all three major central nervous system lineages *in vitro*, the neuronal differentiation of BMSCs *in vivo* is not efficient [25,53,54].

According to recent studies, grafted BMSCs could generate a favorable environment for the regeneration or the differentiation of endogenous NSCs. Previous studies have shown that the transplantation of BMSCs could reduce glial cells and induce axonal regrowth of an injured spinal cord. This positive effect might occur partly because the transplanted BMSCs might have an effect on the oligodendroglial differentiation of endogenous NSCs—promoting NSC differentiation toward oligodendrocytes and preventing NSC differentiation into astrocytes [25,55–57]. Similarly, in our study, the exposure of NSCs to BMSCs resulted in an increase in GalC positive cells and a reduction in GFAP positive cells. Moreover, BMSCs could reverse the BMP4 effect on NSCs by switching the differentiation of NSCs from an astroglial to an oligodendroglial phenotype. These data support a theory that BMSCs were probably capable of providing a favorable environment for the differentiation of endogenous NSCs by reversing the effect of the BMPs that up-regulate after a spinal cord injury. In addition, our results also revealed that Smad1/5 and p-smad1/5, the down-streaming target protein of BMP4, could be inhibited by BMSC-CM. This suggests that BMSCs exert their effect on NSCs by inhibiting the BMP/Smad signaling pathway.

At the molecular mechanism level, we first investigated the expression of Smad1/5 and p-Smad1/5, the down-streaming target protein of BMP4. The result showed that the expression of both Smad1/5 and p-Smad1/5 was up-regulated by the treatment of BMP4, and this up-regulation could be inhibited by the addition of BMSC-CM. This result was consistent with previous findings. Fang et al. suggested that BMSC-CM could decrease the generation of astrocytes with the increasing of neurons via inhibition of the BMP/Smad signaling pathway [58,59]. Then, we examined the expression of Smad1/5 and p-Smad1/5 at early time points (1,6,12, and 24 h) after BMP4 or BMP4+BMSC-CM treatment. We found that Smad1/5 and p-Smad1/5 expression increase after 6 h BMP4 treatment. These proteins were reduced by BMSC-CM in the presence of BMP4 at 6 h and subsequent time points. It indicated that BMSC-CM could exert their effect on NSCs by inhibiting the expression of the down-streaming target protein of BMP4. We also found that after 6 h of BMSC-CM treatment in the presence of BMP4, the expression of Smad1/5 was higher compared with the control groups (*P*<0.05, Figure 4A,B). However, no significant difference of the p-Smad1/5 expression was noted between control groups and 6 h co-culture groups (*P*>0.05, Figure 4A,B). The increasing expression of p-Smad1/5 was observed till 12 h after treatment. This might because that BMSC-CM could affect the process of phosphorylation of Smad protein, resulting in the reduction in p-Smad1/5 expression (As we have mentioned before, BMPs exerted their activity by blinding to Type I and II BMP receptors and formed a heterotetrameric complex. The Smad1/5 proteins were phosphorylated via this heterotetrameric complex).

A large number of studies have reported that both BMPs and BMSCs could determine the fate decision of NSCs by the regulation of Id2 and Olig1/2 expression. For example, the up-regulation of BMP4 could increase the expression of Id2 in endothelial progenitor cells [55]. Similarly, Srikanth et al. revealed that BMP signaling could inhibit oligodendrogliogenesis and promote astrogliogenesis in oligodendroglioma-propagating cells, probably through the inhibition of OLIG1/2 by ID proteins [56]. In contrast, MSC-CM appear to reduce Id2 in proliferating NSCs and, simultaneously, to increase the proportion of Olig2 expression [57]. Another study found that MSC-derived factors were able to accelerate and to promote oligodendroglial differentiation. Moreover, this result was accompanied by the down-regulation of Id2 and Id4 [60]. Francisco et al. also revealed a similar result: an increased percentage of Olig2-expressing cells and a down-regulated Id2 mRNA expression in the 7-day MSC-CM-treated NSCs compared with control [28]. All these studies revealed that both BMPs and MSCs could mediate the expression of Id2 or Olig1/2, but in an opposite mechanism. BMPs could up-regulate Id2 expression with the reduction in Olig1/2 expression, while MSCs were able to down-regulate Id2 expression with the increase in Olig1/2 expression. Our results were consistent with these data. NSCs incubated with BMPs had the highest expression of Id2 and the lowest expression of Olig1 and Olig2. Incubation with BMSC-CM revealed the lowest expression of Id2 with the highest expression of Olig1 and Olig2. Additionally, after the addition of BMSC-CM to NSCs in the presence of BMP4, the Id2 gene expression was decreased and the Olig1/2 gene expression was increased compared with BMP4 groups. This result proved that the BMSC-CM exerted its effect on the mediation of Id2, Olig1, and Olig2 expression in NSCs via blocking the effect of BMP4.

The analysis of the gene expression in early time points after treatment with BMP4 alone or BMP4 and BMSC-CM showed that the BMSC-CM could inhibit the effect of BMP4 on NSCS at 6 h and subsequent time points. Moreover, the result also revealed that these gene changes were consistent with the change of p-Smad1/5 protein in the early time points. In BMP groups, both of these gene expressions and p-Smad1/5 protein expressions were altered significantly after 6 h BMP4 treatment. After the addition of BMSC-CM in co-culture groups, only modest changes of p-Smad1/5 were observed till 12-h treatment. Similarly, these gene expressions were not significant after 12-h treatment in co-culture groups. These results reflected not only that BMSC-CM could reversed the effect of BMP4 as early as 6-h treatment, but also that the expressions of Id2, Olig1, and Olig2 were directly interacting with p-Smad1/5 protein expression. In summary, all these finding indicated that BMSC-CM mediates the Id2, Olig1, and Olig2 expression via the inhabitation of Smad proteins expression.

We also had an interesting finding. To completely block the BMP-signaling pathway, we added Noggin to the control, the BMP-4, and the BMSC-CM groups. The results showed that although nearly no expression of Smad1 and Smad5 was found among these three groups, the BMSC-CM groups still had a lower Id2 expression and a higher Olig1 and Olig2 expression compared with the other two groups. The results indicate that the effect of BMSC-CM on the regulation of Id/Olig balance was not entirely dependent on the BMP/Smad signaling. It might have an effect on some other signaling pathway by which the BMSC-CM could directly moderate the differentiation determination of NSCs. For example, Zhao et al. [61] found that the intravenous administration of MSCs could promote hippocampal neurogenesis in mice with traumatic brain injuries by activating the Wnt/β-catenin signaling pathway, which is considered to play a key role in regulating oligodendrogenesis [62]. The Notch signaling pathway was shown to have a relationship with the regulation of oligodendrocyte progenitor differentiation in adult center neural systems [63]. Moreover, recent studies have found that the co-culture of human NSCs with human MSCs promotes the biology of human NSCs through the activation of the Notch1 pathway [64,65]. Therefore, we thought the effect of MSCs on the fate decision of NSCs was not entirely dependent on the BMP signaling pathway. Some other pathway might also play a role in the process of regulating the differentiation of NSCs. However, this was not proved by the present study and still needs further exploration.

## Conclusion

BMSCs can promote the differentiation of NSCs into oligodendrocytes through mediating Id2 and Olig1/2 expression by blocking the BMP/Smad signaling pathway.

## Abbreviations

bHLH: basic-helix–loop–helix
BMP: bone morphogenetic protein
BMSC: bone marrow stromal cell
BMSC-CM: BMSC-conditioned media
MSC: mesenchymal stem cell
NSC: neural stem cell
SCI: spinal cord injury

## References

[1] Ping K.Y. and Malaspina A. (2012) Spinal cord trauma and the molecular point of no return. Mol. Neurodegener. 7, 1–10 22315999 10.1186/1750-1326-7-6 PMC3299607

[2] Pearse D.D. and Bunge M.B. (2006) Designing cell- and gene-based regeneration strategies to repair the injured spinal cord. J. Neurotrauma 23, 438 10.1089/neu.2006.23.437 16629628

[3] Dietz V. and Curt A. (2006) Neurological aspects of spinal-cord repair: promises and challenges. Lancet Neurol. 5, 688–694 10.1016/S1474-4422(06)70522-1 16857574

[4] Reubinoff B.E., Itsykson P., Turetsky T., Pera M.F., Reinhartz E., Itzik A. (2001) Neural Progenitors from Human Embryonic Stem Cells. Nat. Biotechnol. 19, 1134–1140 10.1038/nbt1201-1134 11731782

[5] Li J. and Lepski G. (2013) Cell transplantation for spinal cord injury: a systematic review. Biomed. Res. Int. 2013, 786475 23484157 10.1155/2013/786475 PMC3581246

[6] Sandner B., Rivera F.J., Caioni M., NicholsonL L.S., Eckstein V., Bogdahn U. (2013) Bone morphogenetic proteins prevent bone marrow stromal cell-mediated oligodendroglial differentiation of transplanted adult neural progenitor cells in the injured spinal cord. Stem Cell Res. 11, 758 10.1016/j.scr.2013.05.003 23770801

[7] Sandner B., Prang P., Rivera F.J., Aigner L., Blesch A., Weidner N. (2012) Neural stem cells for spinal cord repair. Cell Tissue Res. 349, 349 10.1007/s00441-012-1363-2 22388657

[8] Rueger M.A., Muesken S., Walberer M., Jantzen S.U., Schnakenburg K., Backes H. (2012) Effects of minocycline on endogenous neural stem cells after experimental stroke. Neuroscience 215, 174–183 10.1016/j.neuroscience.2012.04.036 22542871

[9] Bonaguidi M.A., Mcguire T., Hu M., Kan L., Samanta J. and Kessler J.A. (2005) LIF and BMP signaling generate separate and discrete types of GFAP-expressing cells. Development 132, 5503 10.1242/dev.02166 16314487

[10] Cheng X., Wang Y., He Q., Qiu M., Whitthemore S.R., Cao Q. (2007) Bone morphogenetic protein signaling and olig1/2 interact to regulate the differentiation and maturation of adult oligodendrocyte precursor cells. Stem Cells 25, 3204–3214 10.1634/stemcells.2007-0284 17872503 PMC2742907

[11] Wang Y., Cheng X., He Q., Zhang Y., Kim D.H., Whitthemore S.R. (2011) Astrocytes from the contused spinal cord inhibit oligodendrocyte differentiation of adult oligodendrocyte precursor cells by increasing the expression of bone morphogenetic proteins. J. Neurosci. Off. J. Soc. Neurosci. 31, 6053–6058 10.1523/JNEUROSCI.5524-09.2011 PMC3081104 21508230

[12] Mabie P.C., Mehler M.F., Marmur R., Papavasiliou A., Song Q. and Kessler J.A. (1997) Bone morphogenetic proteins induce astroglial differentiation of oligodendroglial-astroglial progenitor cells. J. Neurosci. Off. J. Soc. Neurosci. 17, 4112 10.1523/JNEUROSCI.17-11-04112.1997 PMC6573548 9151728

[13] Matsuura I., Taniguchi J., Hata K., Saeki N. and Yamashita T. (2008) BMP inhibition enhances axonal growth and functional recovery after spinal cord injury. J. Neurochem. 105, 1471–1479 10.1111/j.1471-4159.2008.05251.x 18221366

[14] Mctigue D.M., Wei P. and Stokes B.T. (2001) Proliferation of NG2-positive cells and altered oligodendrocyte numbers in the contused rat spinal cord. J. Neurosci. Off. J. Soc. Neurosci. 21, 3392 10.1523/JNEUROSCI.21-10-03392.2001 PMC6762495 11331369

[15] Willerth S.M. and Sakiyama-Elbert S.E. (2008) Cell therapy for spinal cord regeneration. Adv. Drug Deliv. Rev. 60, 263–276 10.1016/j.addr.2007.08.028 18029050 PMC2225623

[16] Prockop D.J. (1997) Marrow Stromal cells as stem cells for nonhematopoietic tissues. Science 276, 71–74 10.1126/science.276.5309.71 9082988

[17] Friedenstein A.J., Deriglasova U.F., Kulagina N.N., Panasuk A.F., Rudakowa S.F., Luriá E.A. (1974) Precursors for fibroblasts in different populations of hematopoietic cells as detected by the in vitro colony assay method. Exp. Hematol. 2, 83 4455512

[18] Pittenger M.F., Mackay A.M., Beck S.C., Jaiswal R.K., Douglas R., Mosca J.D. (1999) Multilineage potential of adult human mesenchymal stem cells. Science 284, 143–147 10.1126/science.284.5411.143 10102814

[19] Brazelton T.R., Rossi F.M., Keshet G.I. and Blau H.M. (2000) From marrow to brain: expression of neuronal phenotypes in adult mice. Science 290, 1775–1779 10.1126/science.290.5497.1775 11099418

[20] Mezey E., Chandross KJ, Harta G., Maki R.A. and Mckercher S.R. (2000) Turning blood into brain: cells bearing neuronal antigens generated in vivo from bone marrow. Science 290, 1779–1782 10.1126/science.290.5497.1779 11099419

[21] Gu W., Zhang Q., Xue Q., Ma Z., Lu P. and Yu B. (2010) Transplantation of bone marrow mesenchymal stem cells reduces lesion volume and induces axonal regrowth of injured spinal cord. Neuropathology 30, 205–217 10.1111/j.1440-1789.2009.01063.x 19845866

[22] Zhang X., Hirai M., Cantero S., Ciubotariu R., Dobrila L., Hirsh A. (2011) Isolation and characterization of mesenchymal stem cells from human umbilical cord blood: reevaluation of critical factors for successful isolation and high ability to proliferate and differentiate to chondrocytes as compared to mesenchymal stem cells from bone marrow and adipose tissue. J. Cell. Biochem. 112, 1206–1218 10.1002/jcb.23042 21312238

[23] Malgieri A., Kantzari E., Patrizi M.P. and Gambardella S. (2010) Bone marrow and umbilical cord blood human mesenchymal stem cells: state of the art. Int. J. Clin. Exp. Med. 3, 248–269 21072260 PMC2971538

[24] Sekiya I., Larson B.L., Smith J.R., Pochampally R., Cui J.G. and Prockop D.J. (2002) Expansion of human adult stem cells from bone marrow stroma: conditions that maximize the yields of early progenitors and evaluate their quality. Stem Cells 20, 530 10.1634/stemcells.20-6-530 12456961

[25] Boido M., Garbossa D., Fontanella M., Ducati A. and Vercelli A. (2014) Mesenchymal Stem Cell Transplantation Reduces Glial Cyst and Improves Functional Outcome After Spinal Cord Compression. World Neurosurgery 81, 183 10.1016/j.wneu.2012.08.014 23022648

[26] Mothe A.J., Bozkurt G., Catapano J., Zabojova J., Wang X., Keating A. (2011) Intrathecal transplantation of stem cells by lumbar puncture for thoracic spinal cord injury in the rat. Spinal Cord 49, 967–973 10.1038/sc.2011.46 21606931

[27] Gu W., Zhang F., Xue Q., Ma Z., Lu P. and Yu B. (2010) Transplantation of bone marrow mesenchymal stem cells reduces lesion volume and induces axonal regrowth of injured spinal cord. Neuropathology 30, 205–217 10.1111/j.1440-1789.2009.01063.x 19845866

[28] Rivera F.J., Couillard-Despres S., Pedre X., Ploetz S., Caioni S., Lois C. (2010) Mesenchymal Stem cells instruct oligodendrogenic fate decision on adult neural stem cells. Stem Cells 24, 2209–2219 10.1634/stemcells.2005-0614 16763198

[29] Cantinieaux D., Quertainmont R., Blacher S., Rossi L., Wanet T., Noël A. (2013) Conditioned medium from bone marrow-derived mesenchymal stem cells improves recovery after spinal cord injury in rats: an original strategy to avoid cell transplantation. PLoS One 8, e69515 10.1371/journal.pone.0069515 24013448 PMC3754952

[30] Chojnacki A. and Weiss S. (2008) Production of neurons, astrocytes and oligodendrocytes from mammalian CNS stem cells. Nat. Protoc. 3, 935–940 10.1038/nprot.2008.55 18536641

[31] Bak S.W., Choi H., Park H.H., Lee K Y., Lee J.L., Yoon M.Y. , Neuroprotective effects of acetyl-L-carnitine against oxygen-glucose deprivation-induced neural stem cell death. Mol. Neurobiol. 2015, 1–9 10.1007/s12035-015-9563-x 26643543

[32] Zhou Q., Wang S. and Anderson D.J. (2000) Identification of a novel family of oligodendrocyte lineage-specific basic helix-loop-helix transcription factors. Neuron 25, 331–343 10.1016/S0896-6273(00)80898-3 10719889

[33] Lu Q.R., Cai L., Rowitch D., Cepko C.L. and Stiles C.D. (2001) Ectopic expression of Olig1 promotes oligodendrocyte formation and reduces neuronal survival in developing mouse cortex. Nat. Neurosci. 4, 973–974 10.1038/nn718 11574831

[34] Zhou A. (2002) The bHLH transcription factors OLIG2 OLIG1 couple neuronal and glial subtype specification. Cell 109, 61–73 10.1016/S0092-8674(02)00677-3 11955447

[35] Hwang D.H., Kim B.G., Kim E.J., Lee S.I., Joo I.S., Suhkim H. (2009) Transplantation of human neural stem cells transduced with Olig2 transcription factor improves locomotor recovery and enhances myelination in the white matter of rat spinal cord following contusive injury. BMC Neuroscience 10, 1–16 10.1186/1471-2202-10-117 19772605 PMC2758886

[36] Cao Y., Liu X., Deng W., Zhang W., Liu Y., Chen L. (2009) TGF-β repression of Id2 induces apoptosis in gut epithelial cells. Oncogene 28, 1089 10.1038/onc.2008.456 19137015 PMC2943843

[37] Sandner B., RiveraF F.J., Caioni M., Nicholson L.S., Eckstein V., Bogdahn U. (2013) Bone morphogenetic proteins prevent bone marrow stromal cell-mediated oligodendroglial differentiation of transplanted adult neural progenitor cells in the injured spinal cord. Stem Cell Res. 11, 758–771 10.1016/j.scr.2013.05.003 23770801

[38] Samanta J. and Kessler J.A. (2004) Interactions between ID and OLIG proteins mediate the inhibitory effects of BMP4 on oligodendroglial differentiation. Development 131, 4131 10.1242/dev.01273 15280210

[39] Grinspan J.B., See J., Bannerman P., Pleasure D. and Ara J., Induction of bone morphogenetic proteins in mouse spinal cord during experimental autoimmune encephalomyelitis. J. Neurochem. 2005, 68–68 10.1002/jnr.21462 17722066

[40] Cheng X., Wang Y., He Q., Qiu M., Whittemore S.R. and Cao Q. (2007) Bone morphogenetic protein signaling and olig1/2 interact to regulate the differentiation and maturation of adult oligodendrocyte precursor cells. Stem Cells 25, 3204–3214 10.1634/stemcells.2007-0284 17872503 PMC2742907

[41] Abe J. (2006) Bone morphogenetic protein (BMP) family, SMAD signaling and Id helix-loop-helix proteins in the vasculature: the continuous mystery of BMPs pleotropic effects. J. Mol. Cell. Cardiol. 41, 4–7 10.1016/j.yjmcc.2006.04.012 16762360

[42] Ten D.P., Korchynskyi O., Valdimarsdottir G. and Goumans M.J. (2003) Controlling cell fate by bone morphogenetic protein receptors. Mol. Cell Endocrinol. 211, 105 10.1016/j.mce.2003.09.016 14656483

[43] Veerasamy M., Phanish M. and Dockrell M.E.C. (2013) Smad mediated regulation of inhibitor of DNA binding 2 and its role in phenotypic maintenance of human renal proximal tubule epithelial cells. PLoS One 8, e51842 10.1371/journal.pone.0051842 23320068 PMC3540025

[44] Tania N.P., Maarsingh H., Is T.B., Mattiotti A., Prakash S., Timens W. (2017) Endothelial follistatin-like-1 regulates the postnatal development of the pulmonary vasculature by modulating BMP/Smad signaling. Pulmonary Circ. 7, 219 10.1177/2045893217702340 28680581 PMC5448549

[45] Dörpholz G., Murgai A., Jatzlau J., Horbelt D., Belverdi M.P., Heroven C. (2017) IRS4, a novel modulator of BMP/Smad and Akt signalling during early muscle differentiation. Sci. Rep. 7, 8778 10.1038/s41598-017-08676-6 28821740 PMC5562708

[46] Nakashima K., Takizawa T., Ochiai W., Yanagisawa M., Hisatsune T., Nakafuku M. (2001) BMP2-mediated alteration in the developmental pathway of fetal mouse brain cells from neurogenesis to astrocytogenesis. Proc. Natl. Acad. Sci. U.S.A. 98, 5868 10.1073/pnas.101109698 11331769 PMC33305

[47] Carrade D.D., Affolter V.K., Outerbridge C.S., Watson J.L., Galuppo L.D., Buerchler S. (2011) Intradermal injections of equine allogeneic umbilical cord-derived mesenchymal stem cells are well tolerated and do not elicit immediate or delayed hypersensitivity reactions. Cytotherapy 13, 1180 10.3109/14653249.2011.602338 21899391

[48] Krampera M., Glennie S., Dyson J., Scott D., Laylor R., Simpson E. (2003) Bone marrow mesenchymal stem cells inhibit the response of naive and memory antigen-specific T cells to their cognate peptide. Blood 101, 3722–3729 10.1182/blood-2002-07-2104 12506037

[49] Keating A. (2006) Mesenchymal stromal cells. Curr. Opin. Hematol. 13, 419 10.1097/01.moh.0000245697.54887.6f 17053453 PMC3365862

[50] Mothe A.J., Bozkurt G., Catapano J., Zabojova J., Wang X., Kealing A. (2011) Intrathecal transplantation of stem cells by lumbar puncture for thoracic spinal cord injury in the rat. Spinal Cord 49, 967–973 10.1038/sc.2011.46 21606931

[51] Gu W., Zhang F., Xue Q., Ma Z., Lu P. and Yu B. (2010) Transplantation of bone marrow mesenchymal stem cells reduces lesion volume and induces axonal regrowth of injured spinal cord. Neuropathology 30, 205–217 10.1111/j.1440-1789.2009.01063.x 19845866

[52] Horky L.L., Galimi F., Gage F.H. and Horner P.J. (2006) Fate of endogenous stem/progenitor cells following spinal cord injury. J. Comp. Neurol. 498, 525–538 10.1002/cne.21065 16874803 PMC2553041

[53] Mctigue D.M., Wei P. and Stokes B.T. (2001) Proliferation of NG2-positive cells and altered oligodendrocyte numbers in the contused rat spinal cord. J. Neurosci. Off. J. Soc. Neurosci. 21, 3392 10.1523/JNEUROSCI.21-10-03392.2001 PMC6762495 11331369

[54] Xu Y., Kitada M., Yamaguchi M., Dezawa M. and Ide C. (2006) Increase in bFGF-responsive neural progenitor population following contusion injury of the adult rodent spinal cord. Neurosci. Lett. 397, 174 10.1016/j.neulet.2005.12.051 16406666

[55] Xia W.H., Chen L., Liang J.W., Zhang X.Y., Su C., Tong X. (2016) BMP4/Id2 signaling pathway is a novel therapeutic target for late outgrowth endothelial progenitor cell-mediated endothelial injury repair. Int. J. Cardiol. 228, 796 10.1016/j.ijcard.2016.11.027 27888757

[56] Srikanth M., Kim J., Das S. and Kessler J.A. (2014) BMP Signaling induces astrocytic differentiation of clinically-derived oligodendroglioma propagating cells. Mol. Cancer Res. Mcr 12, 283 10.1158/1541-7786.MCR-13-0349 24269952 PMC4006982

[57] Steffenhagen C., Dechant F X., Oberbauer E., Furtner T., Weidner N., Kury P. (2012) Mesenchymal stem cells prime proliferating adult neural progenitors toward an oligodendrocyte fate. Stem Cells Dev. 21, 1838 10.1089/scd.2011.0137 22074360 PMC3396148

[58] Fang H., Song P., Shen Y., Shen C. and Liu X., Bone mesenchymal stem cell-conditioned medium decreases the generation of astrocytes during the process of neural stem cells differentiation. J. Spinal Cord. Med. 2017, 1–7 10.1080/10790268.2017.1314880 PMC5810792 28649933

[59] Huang F., Song P.W., Shen C.L., Liu X.Y. and Li H.T. (2016) Bone mesenchymal stem cell-conditioned medium induces the upregulation of Smad6, which inhibits the BMP-4/Smad1/5/8 signaling pathway. Neurol. Res. 38, 965–972 10.1080/01616412.2016.1232549 27636090

[60] Jadasz J.J., Kremer D., Göttle P., Tzekova N., Domke J., Rivera F.J. (2013) Mesenchymal stem cell conditioning promotes rat oligodendroglial cell maturation. PLoS One 8, e71814 10.1371/journal.pone.0071814 23951248 PMC3741203

[61] Zhao Y., Gibb S.L., Zhao J., Moore A.N., Hylin M.J., Menge T. (2016) Wnt3a, a protein secreted by mesenchymal stem cells is neuroprotective and promotes neurocognitive recovery following traumatic brain injury. Stem Cells 34, 1263 10.1002/stem.2310 26840479

[62] Azim K., Fischer B., Hurtado-Chong A., Draganova K., Cantu C., Zemke M. (2014) Persistent Wnt/β-catenin signaling determines dorsalization of the postnatal subventricular zone and neural stem cell specification into oligodendrocytes and glutamatergic neurons. Stem Cells 32, 1301–1312 10.1002/stem.1639 24449255

[63] John G.R., Shankar S.L., Shafitzagardo B., Massimi A., Lee S.C., Raine C.S. (2002) Multiple sclerosis: re-expression of a developmental pathway that restricts oligodendrocyte maturation. Nat. Med. 8, 1115–1121 10.1038/nm781 12357247

[64] Seifert T., Bauer J., Weissert R., Fazekas F. and Storch M.K., Notch1 and its ligand Jagged1 are present in remyelination in a T-cell- and antibody-mediated model of inflammatory demyelination. Acta Neuropathol. 113, 195–203 10.1007/s00401-006-0170-9 17136549

[65] Haragopal H., Yu D., Zeng X., Kim S.W., Han I.B., Ropper A.E. (2015) Stemness enhancement of human neural stem cells following bone marrow MSC co-culture. Cell Transplant. 24, 10.3727/096368915X687561 25719952

